# Percutaneous Coronary Interventions with Sirolimus-Eluting Alex Plus Stents in Patients with or without Diabetes: 4-Year Results

**DOI:** 10.3390/jcdd11060160

**Published:** 2024-05-22

**Authors:** Jacek Bil, Maciej Tyczynski, Adam Kern, Krystian Bojko, Robert J. Gil

**Affiliations:** 1 Department of Invasive Cardiology, Centre of Postgraduate Medical Education, 01-813 Warsaw, Poland; maciej.tyczynski@cskmswia.gov.pl; 2 Department of Cardiology and Internal Medicine, University of Warmia and Mazury in Olsztyn, 11-041 Olsztyn, Poland; adam.kern@uwm.edu.pl (A.K.); krystian.bojko@uwm.edu.pl (K.B.); 3 Department of Cardiology, Regional Specialist Hospital in Olsztyn, 10-561 Olsztyn, Poland; 4 Department of Cardiology, State Medical Institute of the Ministry of Interior and Administration, 02-507 Warsaw, Poland; robert.gil@cskmswia.gov.pl

**Keywords:** SES, PCI, Alex Plus, target lesion revascularization, in-stent restenosis, thin-strut stent

## Abstract

We characterized the performance, as well as the safety, of a second-generation thin-strut sirolimus-eluting stent with a biodegradable polymer, Alex Plus (Balton, Poland), implanted in patients with type 2 diabetes (DM) with a 4-year follow-up. We defined the primary endpoint as the 48-month rate of major cardiovascular adverse events (MACE), including cardiac death, myocardial infarction (MI), or target lesion revascularization (TLR). The secondary endpoints were all-cause death, cardiac death, MI, and TLR rates at 12, 24, 36, and 48 months. We enrolled 232 patients in whom 282 stents were implanted, including 97 DM and 135 non-DM patients. The mean age of the DM patients was 69.5 ± 10.1 years and females accounted for 30% of the patients. DM patients had higher rates of arterial hypertension (97% vs. 88%, *p* = 0.016), dyslipidemia (86% vs. 70%, *p* = 0.005), prior MI (61% vs. 40%, *p* = 0.002), prior PCI (65% vs. 50%, *p* = 0.020), and prior CABG (14% vs. 5.9%, *p* = 0.029). We recorded statistically significant differences for MACE (HR 1.85, 95% CI 1.01–3.41, *p* = 0.046), cardiac death (HR 4.46, 95% CI 1.44–13.8, *p* = 0.010), and MI (HR 3.17, 95% CI 1.10–9.12, *p* = 0.033), but not for TLR, between DM and non-DM patients in terms of the analyzed endpoints at 4 years. Our study showed that Alex Plus was efficient and safe in a contemporary cohort of real-world DM patients undergoing percutaneous revascularization.

## 1. Introduction

Percutaneous coronary intervention (PCI) in patients with diabetes mellitus (DM) presents unique challenges and considerations due to the heightened cardiovascular risk associated with this condition. Diabetes is a significant risk factor for the development and progression of coronary artery disease (CAD), leading to an increased prevalence of complex lesions and a higher likelihood of adverse cardiovascular events. Therefore, managing CAD in DM patients often necessitates more aggressive treatment strategies, including PCI or coronary artery bypass grafting (CABG) [1,2].

One of the key concerns regarding PCI for DM patients is the increased risk of restenosis and stent thrombosis compared to non-DM patients [3]. This heightened risk is attributed to factors such as endothelial dysfunction, accelerated atherosclerosis, and impaired vascular healing processes observed in diabetic patients [4,5]. To mitigate these risks, meticulous attention is paid to the selection of stent types, antiplatelet therapy, and procedural techniques. Drug-eluting stents (DES) are preferred over bare-metal stents (BMS) due to their superior efficacy in reducing restenosis rates [6,7,8]. Moreover, the duration of dual antiplatelet therapy (DAPT) may be extended in DM patients to minimize the risk of stent thrombosis, or more potent antiplatelet drugs might be used, such as ticagrelor [9].

Furthermore, managing DM patients undergoing PCI involves a multidisciplinary approach, with close collaboration between cardiologists, endocrinologists, and other healthcare providers [10]. Optimal glycemic control, along with aggressive management of other cardiovascular risk factors such as hypertension and dyslipidemia, plays a crucial role in improving outcomes post-PCI [6,11]. Additionally, careful patient selection, thorough pre-procedural evaluation, and personalized treatment plans are essential for optimizing PCI outcomes and reducing the risk of complications in DM patients with CAD [12]. Current guidelines recommend CABG for patients with diabetes; however, the diffuse atherosclerotic process in DM patients makes PCI a cornerstone of treatment for DM patients with CAD [13,14].

We characterized the performance and safety of PCI with a second-generation, thin-strut sirolimus-eluting stent (SES) implanted in DM patients, with a 4-year follow-up.

## 2. Materials and Methods

### 2.1. Study Design and Study Population

We retrospectively collected data from the hospital records. We considered all consecutive patients who were subject to PCI with the sirolimus-eluting coronary stent Alex Plus (Balton, Poland) between July 2015 and March 2016, as described previously [15,16].

We analyzed baseline demographics, clinical and laboratory data, and clinical outcomes at a 48-month follow-up between DM and non-DM patients.

### 2.2. Alex Plus Stent Characteristics

Alex Plus is a cobalt–chromium (L605) stent with 70 μm struts that releases sirolimus (1.3 μg/mm^2^) from a biodegradable polymer for eight weeks [17,18]. The Alex Plus stent is available in the following parameter ranges: diameter of 2.0–5.0 mm and length of 8.0–40.0 mm. The stent can be overexpanded during postdilatation even up to 6 mm (3.5 mm -> 4.3 mm; 4.0 mm -> 4.7 mm; 5.0 mm -> 6.0 mm).

### 2.3. Data Collection

We acquired information on the following comorbidities from hospital records: arterial hypertension, diabetes, dyslipidemia, previous myocardial infarction (MI), prior percutaneous coronary intervention (PCI), chronic kidney disease (eGFR < 60 mL/min/1.73m^2^), history of CABG, peripheral artery disease, previous stroke, smoking, and chronic obstructive pulmonary disease. Moreover, we analyzed procedure details, including lesion characteristics (A, B1, B2, C according to AHA/ACC classification [19]) and periprocedural adverse events. Additionally, SYNTAX (https://syntaxscore.org accessed on 24–25 March 2024), SYNTAX II [20], and EuroScore II (https://www.euroscore.org accessed on 19–20 March 2024) were calculated. We also analyzed laboratory results obtained at admission, including complete blood count with differential (WBC—white blood cells, RBC—red blood cells, Hgb—hemoglobin, PLT—platelets), glucose, glycated hemoglobin (HbA1c), troponin T, kinase creatinine (CK), CK-MB, lipid profile, creatinine, and estimated glomerular filtration rate (eGFR). Finally, we summarized the medications prescribed at discharge [14].

Echocardiographic parameters (left ventricular ejection fraction [LVEF], left ventricular end-diastolic diameter, posterior wall diameter, intraventricular septal diameter, tricuspid annular plane systolic excursion, left atrial diameter) were measured with a commercially available diagnostic ultrasound device (iE 33, Philips Medical System, Amsterdam, The Netherlands). Experienced cardiologists measured the values according to the European Association of Cardiovascular Imaging guidelines [21].

### 2.4. Study Endpoints

The primary endpoint of our study was the occurrence rate of major cardiovascular adverse events (MACE) over 48 months, encompassing cardiac death, myocardial infarction (MI), or target lesion revascularization (TLR). Secondary endpoints included rates of all-cause death, cardiac death, MI, and TLR at 12, 24, 36, and 48 months.

### 2.5. Statistical Methods

Descriptive statistics are shown as mean values with standard deviation, minimum values, median values with interquartile range, and maximum values for continuous variables, and categorical variables are presented as counts and percentages. Pearson’s chi-squared test or Fisher’s exact test were employed to compare categorical variables between two subgroups (DM vs. non-DM patients). Fisher’s exact test was used when at least one subgroup had a count of zero. Continuous variables between the two subgroups were compared using the Wilcoxon rank-sum test. A *p*-value < 0.05 was considered statistically significant.

Propensity score matching with the nearest neighbor method was used to adjust for baseline differences. The validity of logistic regression was assessed using the Hosmer–Lemeshow goodness-of-fit test. The model was well calibrated (χ^2^ = 4.33; *p* = 0.84). The propensity model yielded a concordance index 0.76 (95% confidence interval [CI]: 0.69–0.82).

We utilized Kaplan–Meier estimators with 95% CI to compare 48-month survival curves for different endpoints between two subgroups (DM vs. non-DM patients). In cases where a specific endpoint recurred within a patient during the 48-month follow-up, the survival time was recorded as the duration until the first occurrence of that event. It should be noted that when analyzing MACE—a composite endpoint—the survival time was defined as the period leading up to the occurrence of the first event among cardiac death, MI, or TLR.

We performed univariable and multivariable Cox regression analyses, employing the Cox proportional hazards model, to evaluate disparities in survival rates among the groups. The multivariable Cox regression model was chosen using stepwise selection, applying a backward elimination algorithm with a significance threshold of 0.1. Subsequently, we reported the outcomes, including the Hazard Ratio (HR) and the corresponding 95% confidence intervals for HR.

Statistical analyses were conducted using R software version 4.2.1 (23 June 2022 ucrt)—“Funny-Looking Kid” copyright 2022, The R Foundation for Statistical Computing, platform: x86_64-w64-mingw32/x64 (64-bit) [14].

## 3. Results

### 3.1. Baseline Characteristics

**Figure 1:**
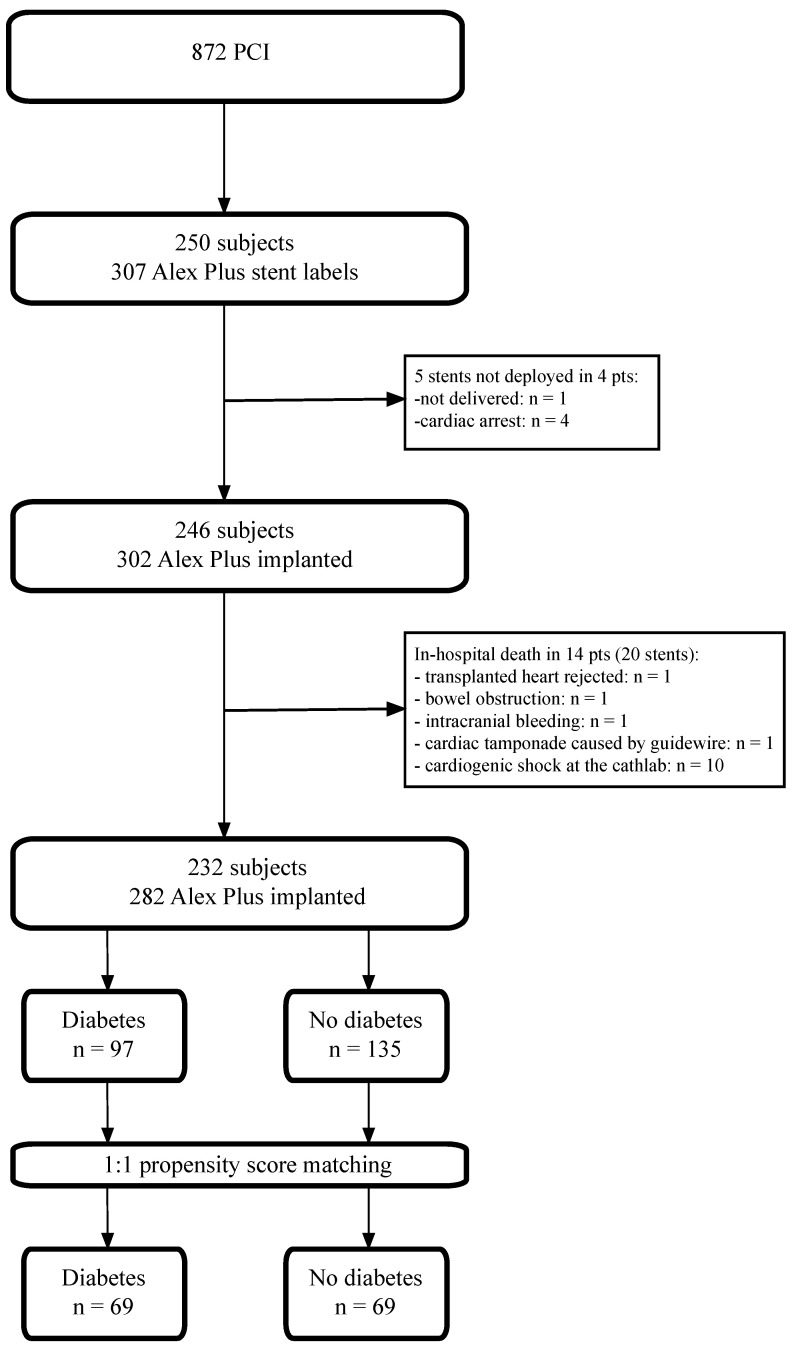
The study flow chart.

In the reporting time frame, we retrieved data on 872 PCI procedures. For the final analysis, we included 232 patients with 282 Alex Plus stents implanted, as described previously in detail. In 4 subjects (5 stents), Alex Plus stents were not deployed (1 device failure—no possibility to deliver the stent to the target lesion due to calcification and tortuosity; 4 stents not implanted due to fatal cardiac arrest). Additionally, 14 subjects were excluded (20 stents) due to in-hospital death unrelated to the sirolimus-eluting stent deployment [15,16,22]. In the end, we identified 97 DM patients and 135 non-DM patients (Figure 1).

The mean age of the DM patients was 69.5 ± 10.1 years, and females accounted for 30% of the patients. The DM patients had higher rates of arterial hypertension (97% vs. 88%, *p* = 0.016), dyslipidemia (86% vs. 70%, *p* = 0.005), prior MI (61% vs. 40%, *p* = 0.002), prior PCI (65% vs. 50%, *p* = 0.020), and prior CABG (14% vs. 5.9%, *p* = 0.029). They were also characterized by higher values in echocardiographic parameters revealing left ventricular hypertrophy (Table 1). Patients with diabetes had higher HbA1c (7.9 ± 9.5% vs. 5.6 ± 1.6%, *p* < 0.001) and triglyceride (175.3 ± 85.7 mg% vs. 117.6 ± 67.5 mg%, *p* = 0.007) values as well as lower eGFR (66.6 ± 22.2 mL/min/1.73m^2^ vs. 73.4 ± 23.6 mL/min/1.73m^2^, *p* = 0.028) values (Table 2).

### 3.2. Procedure Characteristics

We observed no significant differences between DM and non-DM patients, except for treated coronary bifurcation incidence, which was higher in the DM subgroup (19.5% vs. 2.9%, *p* = 0.012). Most treated lesions were located in the right coronary artery (DM vs. non-DM: 40% vs. 37%, *p* = 0.812), followed by the left anterior descending artery (31.0% vs. 30%, *p* = 0.812) and the left circumflex artery (23% vs. 28%, *p* = 0.812). Lesions undergoing PCI were complex. Type C lesions were treated in 35% of the DM cases and 39% of the non-DM cases (*p* = 0.896). The mean SYNTAX score was numerically higher in DM patients (14.3 ± 8.5 vs. 13.6 ± 8.8, *p* = 0.441) (Table 3).

Lesion pre- (66% vs. 59%, *p* = 0.249) and postdilatations (35% vs. 40%, *p* = 0.444) were performed at similar rates. The mean nominal stent parameters did not differ significantly among subgroups. Device success was 100% in the non-DM group and 98.9% in the DM group (one stent failure case occurred due to massive calcification). Additional stents were implanted in 44% of DM patients and 35% of non-DM patients (*p* = 0.155). Coronary dissections were comparable among subgroups (6.2% vs. 7.4%, *p* = 0.717) (Table 3).

Table 4 provides the drugs administered at discharge. All patients received acetylsalicylic acid and P2Y12 inhibitors. Patients with DM more frequently received Ca-blockers, diuretics, and nitrates.

### 3.3. Long-Term Outcomes

The MACE, death, cardiac death, MI, and TLR rates at 12, 24, 36, and 48 months for the whole population were published previously [15]. At 48 months, for the DM patients, the incidences of MACE, death, cardiac death, MI, and TLR were 25.6%, 17.5%, 12.4%, 10.3%, and 12.4%, respectively (Table 5). The reasons for cardiac death were heart failure deterioration (*n* = 10), cardiogenic shock due to MI (*n* = 1), and sudden cardiac death (*n* = 1). No stent thrombosis cases were registered. In the whole population, there were statistically significant differences for MACE (HR 1.85, 95% CI 1.01–3.41, *p* = 0.046), cardiac death (HR 4.46, 95% CI 1.44–13.8, *p* = 0.010), and MI (HR 3.17, 95% CI 1.10–9.12, *p* = 0.033) between DM and non-DM patients at 4 years (Supplementary Figure S1).

Additionally, in Supplementary Figure S2, we present data on all-cause death rates at 8 years. The overall mortality rate was 28% (*n* = 65), with a 39.2% (*n* = 38) mortality rate in the DM subgroup and 20.0% (*n* = 27) in the non-DM subgroup (HR 2.31, 95% CI 1.40–3.81, *p* = 0.01).

### 3.4. 4-Year Outcomes in Propensity Score Matching

Propensity score matching yielded 69 well-matched pairs of patients with DM or without DM. Baseline clinical and procedural differences were balanced (Table 1, Table 2, Table 3 and Table 4).

**Figure 2:**
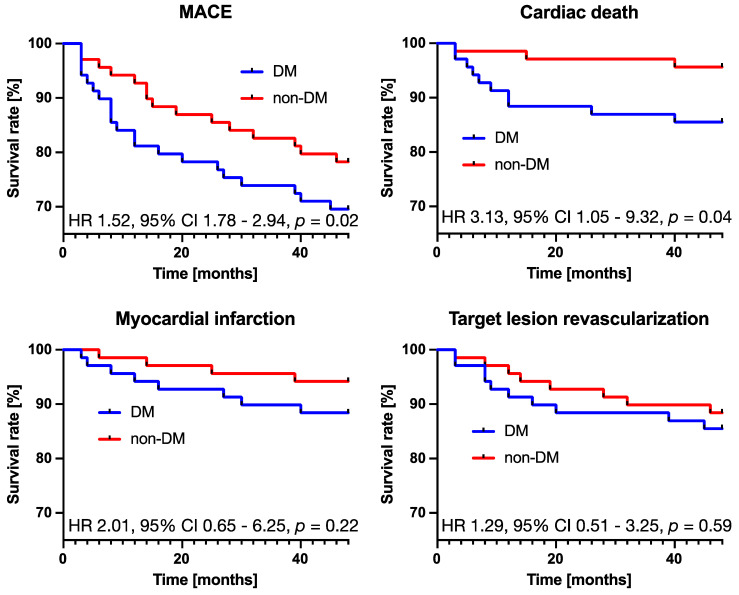
Kaplan–Meier curves disclosing event-free survival in DM and non-DM subgroups in propensity score-matched population. DM—diabetes; MACE—major adverse cardiovascular events.

At 48 months, in the DM patients, the incidences of MACE, death, cardiac death, MI, and TLR were 30.4%, 18.8%, 14.5%, 11.6%, and 14.5%, respectively (Table 6). No stent thrombosis cases were registered. In the propensity score-matched population, there were statistically significant differences for MACE (HR 1.52, 95% CI 1.78–2.94, *p* = 0.02) and cardiac death (HR 3.13, 95% CI 1.05–9.32, *p* = 0.04) between DM and non-DM patients at 4 years (Figure 2).

### 3.5. Cox Analysis

Finally, we analyzed predictive factors for MACE and TLR in the DM subgroup at 48 months. The multivariable analysis results are depicted in Table 7 for MACE and Table 8 for TLR (univariable analyses are presented in Supplementary Tables S1 and S2).

In the multivariable Cox regression analysis, the statistically significant MACE predictors were postdilatation (HR 3.76, 95% CI 1.56–9.08, *p* = 0.003); EuroScore > 3 (HR 5.8, 95% CI 1.92–17.5, *p* = 0.002); arterial hypertension (HR 0.16, 95% CI 0.03–0.78, *p* = 0.023); and clopidogrel use (HR 0.28, 95% CI 0.09–0.93, *p* = 0.038), whereas the TLR predictors were postdilatation (HR 14.5, 95% CI 2.77–75.7, *p* = 0.002); EuroScore > 3 (HR 6.72, 95% CI 1.43–31.5, *p* = 0.016); cardiogenic shock (HR 42.2, 95% CI 2.21–80.5, *p* = 0.013); and clopidogrel use (HR 0.15, 95% CI 0.03–0.71, *p* = 0.017).

## 4. Discussion

Our study demonstrated that Alex Plus stents were effective and safe for a modern group of real-world diabetic patients undergoing percutaneous revascularization. Using Alex Plus for PCI resulted in low rates of periprocedural complications and a high device success rate of nearly 99%. Predictably, diabetic patients experienced higher rates of MACE, cardiac death, and MI over a 4-year period. However, the TLR rates between diabetic and non-diabetic patients did not show a significant statistical difference at the 4-year follow-up.

Diabetes presents unique challenges in the management of coronary artery disease, necessitating tailored approaches to revascularization strategies. Drug-eluting stents (DES) have revolutionized the treatment landscape by significantly reducing restenosis rates and the need for repeat revascularization procedures [23,24,25]. Sirolimus-eluting stents (SES) have emerged as a prominent choice for diabetic patients due to their potent antiproliferative properties and favorable outcomes in inhibiting neointimal hyperplasia, a common complication in this population. Studies have consistently shown improved clinical outcomes with SES compared to bare-metal stents (BMS) and first-generation DES, making them a preferred option in diabetic patients undergoing percutaneous coronary intervention (PCI) [26,27,28].

One of the key advantages of sirolimus-eluting stents in diabetic patients lies in their ability to mitigate the heightened risk of restenosis and adverse events associated with coronary interventions in this population [29]. Diabetes is characterized by systemic inflammation, endothelial dysfunction, and increased proliferation of smooth muscle cells, all of which contribute to accelerated atherosclerosis and restenosis post-PCI [5,30,31]. Sirolimus, a potent immunosuppressant and antiproliferative agent, effectively inhibits smooth muscle cell proliferation and migration, thereby reducing the risk of restenosis. This property is particularly advantageous in diabetic patients, where restenosis rates tend to be higher compared to non-diabetic patients [1,3,32].

Despite the all-comer nature of this research, the incidence of periprocedural complications was low. One potential explanation might be the fact that transradial access was used in over 80% of the patients. Transradial access is acknowledged to be linked with a decreased risk of adverse events compared to femoral access, particularly in high-risk patients [33].

After 12 months, the cardiac death, TLR, MI, and MACE rates for the DM patients were 10.3%, 9.3%, 7.2%, and 17.5%, respectively. These rates increased at 48 months to 12.4%, 12.4%, 10.3%, and 25.6%, respectively. As can be seen, the highest incidence of events was observed in the first 12 months after the index procedure. In our study population, the MACE rate was mainly driven by cardiac death cases associated with heart failure exacerbation. The cardiac death rates in the non-DM patients were markedly lower—0.74% at 12 months, and 2.9% at 48 months. What is somewhat surprising is that the TLR rates were comparable between DM and non-DM patients [34,35]. These results are comparable to those reported in the literature.

Gasior et al. compared biodegradable polymer sirolimus-eluting stents (BP-SES) with durable polymer everolimus-eluting stents (DP-EES) [36]. At 12 months, they observed similar rates of target vessel revascularization (6.64% vs. 5.88%; *p* = 0.611), as well as similar safety outcomes: all-cause death (10.06% vs. 7.59%; *p* = 0.158), MI (7.959% vs. 6.83%; *p* = 0.813), and definite/probable stent thrombosis (1.14% vs. 0.76%; *p* = 0.525). Worth stressing is the fact that in our study, there were no cases of stent thrombosis.

Olsen et al. analyzed 5-year results of DM patients treated with either zotarolimus-eluting stents (ZES) or SES [37]. In these DM patients, the MACE rate was higher in patients treated with ZES than those treated with SES (28.4% vs. 18.5%; *p* = 0.032) due to an increased rate of target vessel revascularization (TVR, 18.9% vs. 8.3%; *p* = 0.006). Among non-DM patients, ZES and SES characterized similar MACE rates at 5 years; however, SES were linked with a significantly increased risk of definite stent thrombosis (1.0% vs. 2.3%; *p* = 0.028).

Conversely, Iglesis et al. showed that the 5-year rates of cardiac death (12.3% vs. 6.8%; *p* < 0.01), target vessel MI (11.2% vs. 5.4%; *p* < 0.01), and clinically-driven TLR (16.4% vs. 8.6%; *p* < 0.01) were significantly higher in DM patients than in non-DM individuals [38].

Here, it is also worth referencing the paper by Koch et al., in which 10-year follow-up data are presented [39]. This study involved a total of 3002 participants who were randomly allocated to receive either polymer-free (PF)-SES (*n* = 2002) or durable polymer (DP)-ZES (*n* = 1000). The DM prevalence was notably high and comparable between the groups, with 575 patients (28.7%) in the PF-SES group and 295 patients (29.5%) in the DP-ZES group (p = 0.66). Over a span of 10 years, the survival rates were 53.5% for the DM patients and 68.5% for the non-DM patients. In terms of MACE, PF-SES demonstrated similar rates as DP-ZES in DM patients (74.8% vs. 79.6%; *p* = 0.08) as well as in non-DM patients (62.5% vs. 62.2%; *p* = 0.88).

In our study, 19.5% of PCI cases were performed within coronary bifurcations in DM patients. In the BIFURCAT registry, DM patients (compared to non-DM patients) undergoing PCI within coronary bifurcations are characterized by worse outcomes (a median follow-up of 21 months) due to increased rates of MACE (17% vs. 9%, *p* < 0.01), all-cause mortality (9% vs. 4%, *p* < 0.01), TLR (5% vs. 3%, *p* < 0.01), and stent thrombosis (2% vs. 1%, *p* < 0.01) [40].

Finally, we identified predicting factors of MACE and TLR. They are well known, and include higher EuroScores and cardiogenic shock. Nevertheless, postdilatation (destructive) and arterial hypertension (protective) had a strikingly high impact. The reason for this is unclear. One might speculate that patients with arterial hypertension were under strict surveillance, with better risk factor control. Also, clopidogrel use seemed to protect against MACE, but one can presume that ticagrelor or prasugrel were preferentially given to patients with more complex lesions. Nonetheless, overly aggressive postdilatation could potentially lead to higher rates of vessel injury, coronary dissection, and neointima response, which, in consequence, could cause ischemic events in the future. In this context, one study showed that the stent’s diameter and length did not impact the outcomes of DM patients [41].

### Study Limitations

This research possesses inherent constraints typical of observational studies, wherein treatment selection relied on the operator’s discretion. The lack of randomization might introduce selection bias, although consecutive patient enrollment somewhat mitigated this concern. Moreover, the relatively modest size of the study cohort and challenges in gathering follow-up information might have influenced the outcomes. Furthermore, the absence of a formal sample size calculation could have impacted the results. To partially correct this, a propensity score matching analysis was performed. Finally, the low rate of intravascular imaging could also negatively impact the results.

## 5. Conclusions

Our study showed that Alex Plus stents were efficient and safe in a contemporary cohort of real-world DM patients undergoing percutaneous revascularization. PCI with Alex Plus was associated with low periprocedural complication rates and high device success (almost 99%). As could be predicted, the rates of MACE, cardiac death, and MI were higher in DM patients at 4 years. However, the TLR rates did not significantly differ statistically between DM and non-DM patients at the 4-year follow-up.

## Figures and Tables

**Table 1:** Baseline characteristics.

	Unmatched Population				Propensity Score-Matched Population		
Variable	Total Population *n* = 232 (%)	DM *n* = 97 (%)	Non-DM *n* = 135 (%)	*p*	DM *n* = 69 (%)	Non-DM *n* = 69 (%)	*p*
Sex: female	64 (27.6)	29 (30)	35 (26)	0.504	19 (27.5)	18 (26.1)	1.0
Age [years]	68 ± 11	69.5 ± 10.1	66.7 ± 11.3	0.071	67.4 ± 9.8	66.9 ± 8.9	0.75
Acute coronary syndrome type at presentation							
Unstable angina	30 (12.9)	13 (13)	17 (13)	0.357	7 (10.1)	7 (10.1)	0.78
Non-ST elevation MI	26 (11.2)	10 (10)	16 (12)		6 (8.7)	7 (10.1)	
ST elevation MI	32 (13.8)	10 (10)	23 (17)		6 (8.7)	8 (11.6)	
Cardiogenic shock	6 (2.6)	2 (2.1)	4 (3.0)	>0.999	2 (3.3)	2 (3.3)	1.0
Arterial hypertension	213 (91.8)	94 (97)	119 (88)	0.016	68 (98.6)	66 (95.7)	0.62
Dyslipidemia	177 (76.3)	83 (86)	94 (70)	0.005	55 (79.7)	54 (78.3)	1.0
Prior myocardial infarction	113 (48.7)	59 (61)	54 (40)	0.002	39 (56.5)	41 (59.4)	0.86
Prior PCI	130 (56.0)	63 (65)	67 (50)	0.020	42 (60.1)	38 (55.1)	0.61
Prior CABG	22 (9.5)	14 (14)	8 (5.9)	0.029	9 (13.0)	7 (10.1)	0.79
Chronic kidney disease	42 (18.1)	21 (22)	21 (16)	0.234	12 (7.2)	11 (15.9)	1.0
Prior stroke	17 (7.3)	8 (8.2)	9 (6.7)	0.649	7 (10.1)	7 (10.1)	1.0
Peripheral artery disease	25 (10.8)	9 (9.3)	16 (12)	0.533	7 (10.1)	8 (11.6)	1.0
Chronic obstructive pulmonary disease	13 (5.6)	8 (8.2)	5 (3.7)	0.138	4 (5.8)	3 (4.3)	1.0
Echocardiographic parameters							
Left ventricular end-diastolic diameter [mm]	50.4 ± 9.0	51.1 ± 6.9	49.9 ± 10.1	0.507	51.0 ± 5.4	50.4 ± 13.2	0.73
Intraventricular septal diameter [mm]	11.4 ± 2.1	11.9 ± 2.1	11.1 ± 2.1	0.017	11.5 ± 4.1	11.2 ± 3.2	0.63
Posterior wall diastolic diameter [mm]	10.5 ± 1.6	10.7 ± 1.5	10.3 ± 1.7	0.043	10.5 ± 1.9	10.3 ± 2.1	0.59
Left atrium [mm]	40.4 ± 5.9	41.6 ± 6.2	39.6 ± 5.5	0.043	41.0 ± 4.1	39.9 ± 7.2	0.27
TAPSE [mm]	22.0 ± 4.3	21.8 ± 4.6	22.1 ± 4.2	0.421	21.9 ± 4.9	22.2 ± 3.5	0.68
LVEF [%]	49.5 ± 10.5	49.0 ± 9.9	49.8 ± 11.0	0.366	49.0 ± 9.9	49.8 ± 11.0	0.65
Severe mitral insufficiency	6 (3.1)	1 (1.3)	5 (4.4)	0.404	1 (1.4)	2 (3.3)	1.0
Severe aortic insufficiency	1 (0.5)	1 (1.3)	0 (0)	0.412	0	0	1.0
Severe aortic stenosis	4 (2.1)	3 (3.8)	1 (0.9)	0.308	0	0	1.0

DM—type 2 diabetes; MI—myocardial infarction; CABG—coronary artery bypass grafting; LVEF—left ventricular ejection fraction; PCI—percutaneous coronary intervention; TAPSE—tricuspid annular plane systolic excursion.

**Table 2:** Laboratory results.

	Unmatched Population				Propensity Score-Matched Population		
Variable	Total Population *n* = 232	DM *n* = 97	Non-DM *n* = 135	*p*	DM *n* = 69	Non-DM *n* = 69	*p*
White blood cells [10^9^/L]	8.5 ± 2.7	8.5 ± 2.7	8.6 ± 2.7	0.67	8.5 ± 1.9	8.5 ± 2.1	1.0
Hemoglobin [g/dL]	13.4 ± 1.7	13.1 ± 1.8	13.6 ± 1.5	0.09	13.1 ± 1.5	13.4 ± 2.2	0.35
Red blood cells [10^12^/L]	4.4 ± 0.5	4.4 ± 0.6	4.5 ± 0.5	0.27	4.4 ± 0.5	4.5 ± 0.4	0.19
Platelets [10^9^/L]	222.9 ± 65	225.1 ± 61.6	221.3 ± 67.6	0.20	224.4 ± 54.3	229.1 ± 61.7	0.64
Glucose [mg/dL]	136.4 ± 64.9	170.8 ± 75.0	110.7 ± 40.5	<0.001	145.1 ± 90.4	124.1 ± 59.3	0.11
HbA1c [%]	6.3 (6.0–7.3)	7.9 ± 9.5	5.6 ± 1.6	<0.001	7.5 ± 4.5	5.9 ± 2.6	0.01
Total cholesterol [mg/dL]	163.9 ± 50.9	161.5 ± 60.6	165.7 ± 42.5	0.24	163.8 ± 43.5	161.9 ± 55.2	0.82
HDL [mg/dL]	45.7 ± 14.6	43.1 ± 12.1	47.6 ± 16.0	0.09	42.1 ± 9.1	45.5 ± 15.5	0.12
LDL [mg/dL]	89.8 ± 40.5	93.2 ± 39.5	85.3 ± 41.6	0.21	87.2 ± 44.2	89.2 ± 55.2	0.81
Triglycerides [mg/dL]	142 ± 33.9	175.3 ± 85.7	117.6 ± 67.5	0.01	154.8 ± 90.4	135.9 ± 70.9	0.17
Creatine [mg/dL]	1.1 ± 0.7	1.2 ± 0.8	1.1 ± 0.6	0.12	1.2 ± 0.6	1.1 ± 0.7	0.37
eGFR [mL/min/1.73 m^2^]	70.5 ± 23.2	66.6 ± 22.2	73.4 ± 23.6	0.03	68.9 ± 32.1	71.9 ± 13.9	0.48

Results presented as mean ± standard deviation; HDL—high-density lipoprotein; LDL—low-density lipoprotein.

**Table 3:** Periprocedural characteristics.

	Unmatched Population				Propensity Score-Matched Population		
Variable	Total Population *n* = 232 (%)	DM *n* = 97 (%)	Non-DM *n* = 135 (%)	*p*	DM *n* = 69 (%)	Non-DM *n* = 69 (%)	*p*
Coronary artery with the target lesion							
LM	9 (3.9)	4 (4.1)	5 (3.7)	0.81	3 (4.3)	3 (4.3)	1.0
LAD	70 (30)	30 (31)	40 (30)		25 (36.2)	29 (42.0)	
LCx	60 (25.9)	22 (23)	38 (28)		17 (24.6)	18 (26.1)	
RCA	89 (38.4)	39 (40)	50 (37)		24 (34.8)	19 (27.5)	
VG	5 (2.2)	3 (3.1)	2 (1.5)		0	0	
Type of target lesion							
A	43 (18.5)	19 (20)	24 (18)	0.89	14 (20.3)	18 (26.1)	0.28
B1	65 (28.0)	29 (30)	36 (27)		19 (27.5)	19 (27.5)	
B2	37 (15.9)	15 (15)	22 (16)		10 (14.5)	12 (17.4)	
C	87 (37.5)	34 (35)	53 (39)		26 (37.7)	19 (27.5)	
Heavy calcification	18 (7.8)	11 (11)	7 (5.2)	0.08	6 (8.7)	5 (7.2)	1.0
Coronary bifurcation	23 (9.9)	19 (19.5)	4 (2.9)	0.01	8 (11.6)	4 (5.8)	0.37
SYNTAX	13.9 ± 8.7	14.3 ± 8.5	13.6 ± 8.8	0.44	14.1 ± 8.8	13.7 ± 7.2	0.40
SYNTAX II PCI	32.9 ± 11.0	33.7 ± 10.5	32.4 ± 11.3	0.31	33.2 ± 10.9	32.5 ± 10.4	0.70
SYNTAX II CABG	29.1 ± 10.8	29.0 ± 11.1	29.1 ± 10.7	0.64	29.2 ± 9.2	29.4 ± 8.9	0.89
EuroScore II	2.9 + 4.3	4.1 ± 6.1	2.5 ± 4.3	0.13	3.4 ± 5.2	2.7 ± 3.5	0.36
Lesion predilatation	143 (61.6)	64 (66)	79 (59)	0.25	40 (57.9)	38 (55.1)	0.86
Stent diameter [mm]	3.2 ± 0.5	3.2 ± 0.5	3.1 ± 0.5	0.91	3.1 ± 0.4	3.1 ± 0.7	1.0
Stent length [mm]	21.2 ± 10.9	25.9 ± 12	17.6 ± 6.5	0.07	22.1 ± 7.8	19.2 ± 8.5	0.04
Stent pressure [atm]	15.3 ± 2.7	15.7 ± 2.6	15.1 ± 2.7	0.09	15.4 ± 2.5	15.8 ± 2.2	0.32
2nd stent implantation	90 (39)	43 (44)	47 (35)	0.16	31 (44.9)	29 (42.0)	0.86
Stent postdilatation	88 (37.9)	34 (35)	54 (40)	0.44	25 (36.2)	27 (39.1)	0.86
Access site							
Transradial	193 (83.2)	81 (84)	112 (83)	0.91	60 (86.9)	58 (84.1)	0.81
Transfemoral	39 (16.8)	16 (16)	23 (17)		9 (13.0)	11 (15.9)	
Guiding catheter *							
6F	222 (95.7)	91 (93.8)	131 (96.3)	0.33	65 (94.2)	67 (97.1)	0.68
7F	11 (4.7)	6 (6.2)	5 (3.7)		4 (5.8)	2 (2.9)	
Intravascular imaging							
IVUS	20 (8.6)	11 (11.3)	9 (6.7)	0.24	5 (7.2)	4 (5.8)	1.0
OCT	8 (3.4)	3 (3.1)	5 (3.7)	0.76	2 (2.9)	2 (2.9)	1.0
Coronary dissection	16 (6.9)	6 (6.2)	10 (7.4)	0.72	4 (5.8)	5 (7.2)	1.0
MI type 4a	5 (2.2)	2 (2.1)	3 (2.2)	>0.99	2 (2.9)	2 (2.9)	1.0

* More than one access or catheter was used during the procedure; DM—diabetes; LM—left main; LAD—left anterior descending artery; LCx—left circumflex artery; MI—myocardial infarction; RCA—right coronary artery; VG—vein graft.

**Table 4:** Drugs administered at discharge.

	Unmatched Population				Propensity Score-Matched Population		
Variable	Total Population *n* = 232 (%)	DM *n* = 97 (%)	Non-DM *n* = 135 (%)	*p*	DM *n* = 69 (%)	Non-DM *n* = 69 (%)	*p*
Acetylsalicylic acid	232 (100)	97 (100)	135 (100)	1.0	69 (100)	69 (100)	1.0
P2Y12							
Clopidogrel	214 (92.2)	89 (92)	125 (93)	0.68	64 (92.3)	64 (92.3)	1.0
Prasugrel	1 (0.4)	1 (1.0)	0 (0)		0	0	
Ticagrelor	17 (7.3)	7 (7.2)	10 (7.4)		5 (7.2)	5 (7.2)	
Beta-blocker	223 (96.1)	96 (99)	127 (94)	0.08	69 (100)	67 (97.1)	0.49
Ca-blocker	53 (22.8)	30 (31)	23 (17)	0.01	16 (23.2)	13 (18.8)	0.68
Angiotensin-converting enzyme inhibitor	190 (81.9)	78 (80)	112 (83)	0.62	56 (81.2)	59 (85.5)	0.65
Angiotensin receptor blocker	36 (15.5)	17 (18)	19 (14)	0.47	11 (15.9)	9 (13.0)	0.81
Diuretic	125 (53.9)	65 (67)	60 (44)	<0.001	40 (57.9)	36 (52.2)	0.61
Mineralocorticoid receptor antagonist	48 (20.7)	17 (18)	31 (23)	0.31	9 (13.0)	11 (15.9)	0.81
Nitrates	13 (5.6)	10 (10)	3 (2.2)	0.01	5 (7.2)	2 (2.9)	0.44
Vitamin K antagonist	17 (7.3)	6 (6.2)	11 (8.1)	0.57	3 (4.3)	4 (5.8)	1.0
Non-vitamin K oral anticoagulant	11 (4.7)	7 (7.2)	4 (3.0)	0.21	3 (4.3)	2 (2.9)	1.0
Statin	230 (99.1)	95 (98)	135 (100)	0.17	69 (100)	69 (100)	1.0
Hypoglycemic medications	62 (26.7)	60 (62)	2 (1.5)	<0.001	45 (65.2)	0	<0.001
Insulin	33 (14.2)	33 (33)	0	<0.001	23 (33.3)	0	<0.001

DM—diabetes.

**Table 5:** Study endpoints by year in patients with diabetes and without diabetes.

Year	Death	Cardiac Death	TLR	MI	MACE	Patient Number
Patients with diabetes						
1	13 (13.4%)	10 (10.3%)	9 (9.3%)	7 (7.2%)	17 (17.5%)	97
2	14 (14.4%)	11 (11.3%)	12 (12.4%)	7 (7.2%)	21 (21.6%)	84
3	15 (15.4%)	12 (12.4%)	12 (12.4%)	7 (7.2%)	22 (22.7%)	83
4	17 (17.5%)	12 (12.4%)	12 (12.4%)	10 (10.3%)	25 (25.6%)	82
Patients without diabetes						
1	4 (2.9%)	1 (0.74%)	9 (6.7%)	2 (1.5%)	10 (7.4%)	135
2	5 (3.7%)	2 (1.5%)	16 (11.9%)	4 (2.9%)	18 (13.3%)	131
3	6 (4.4%)	3 (2.2%)	19 (14.1%)	4 (2.9%)	22 (16.3%)	130
4	8 (5.9%)	4 (2.9%)	22 (16.3%)	6 (4.4%)	29 (21.5%)	129

*n* (%). MACE—major adverse cardiovascular events; MI—myocardial infarction; TLR—target lesion revascularization.

**Table 6:** Study endpoints by year in patients with diabetes and without diabetes (propensity score-matched population).

Year	Death	Cardiac Death	TLR	MI	MACE	Patient Number
Patients with diabetes						
1	9 (13.0%)	8 (11.6%)	6 (8.7%)	4 (5.8%)	13 (18.8%)	69
2	10 (14.5%)	8 (11.6%)	8 (11.6%)	5 (7.2%)	16 (23.2%)	60
3	11 (15.9%)	9 (13.0%)	8 (11.6%)	7 (10.1%)	19 (27.5%)	50
4	13 (18.8%)	10 (14.5%)	10 (14.5%)	8 (11.6%)	21 (30.4%)	39
Patients without diabetes						
1	2 (2.9%)	1 (1.4%)	3 (4.3%)	1 (1.4%)	5 (7.2%)	69
2	3 (4.3%)	2 (2.9%)	5 (7.2%)	2 (2.9%)	9 (13.0%)	67
3	3 (4.3%)	2 (2.9%)	7 (10.1%)	3 (4.3%)	12 (17.4%)	64
4	4 (5.8%)	3 (4.3%)	8 (11.6%)	4 (5.8%)	15 (21.7%)	61

**Table 7:** Multivariable Cox analysis: major adverse cardiovascular events.

Variable	Multivariable Analysis for Major Adverse Cardiovascular Events		
	HR	95% CI	*p*
Postdilatation	3.76	1.56, 9.08	0.003
EuroScore > 3.0	5.80	1.92, 17.5	0.002
Arterial hypertension	0.16	0.03, 0.78	0.023
Clopidogrel use	0.28	0.09, 0.93	0.038

HR—hazard ratio; CI—confidence interval.

**Table 8:** Multivariable Cox analysis: target lesion revascularization.

Variable	Multivariable Analysis for Target Lesion Revascularization		
	HR	95% CI	*p*
Postdilatation	14.5	2.77, 75.7	0.002
EuroScore > 3.0	6.72	1.43, 31.5	0.016
Cardiogenic shock	42.2	2.21, 80.5	0.013
Clopidogrel use	0.15	0.03, 0.71	0.017

HR—hazard ratio; CI—confidence interval.

## Data Availability

Data are available from the corresponding author on request.

## Supplementary Materials

The following supporting information can be downloaded at: https://www.mdpi.com/article/10.3390/jcdd11060160/s1, Supplementary Table S1: Univariable Cox regression for MACE; Supplementary Table S2: Univariable Cox regression for TLR; Supplementary Figure S1: Kaplan–Meier curves disclosing event-free survival in DM and non-DM subgroups in the whole population. Supplementary Figure S2: Kaplan–Meier curves disclosing event-free survival in DM and non-DM subgroups in the whole population at 8 years.

## References

[1] Ma J., Wang M., Wu P., Ma X., Chen D., Jia S., Yan N. (2024). Predictive effect of triglyceride-glucose index on No-Reflow Phenomenon in patients with type 2 diabetes mellitus and acute myocardial infarction undergoing primary percutaneous coronary intervention. Diabetol. Metab. Syndr..

[2] Marx N., Federici M., Schutt K., Muller-Wieland D., Ajjan R.A., Antunes M.J., Christodorescu R.M., Crawford C., Di Angelantonio E., Eliasson B. (2023). 2023 ESC Guidelines for the management of cardiovascular disease in patients with diabetes. Eur. Heart J..

[3] Tanner R., Farhan S., Giustino G., Sartori S., Feng Y., Hooda A., Vinayak M., Dangas G., Mehran R., Kini A.S. (2024). Impact of diabetes mellitus on clinical outcomes after first episode in-stent restenosis PCI: Results from a large registry. Int. J. Cardiol..

[4] Rao C., Zhong Q., Wu R., Li Z., Duan Y., Zhou Y., Wang C., Chen X., Wang R., He K. (2024). Impact of body mass index on long-term outcomes in patients undergoing percutaneous coronary intervention stratified by diabetes mellitus: A retrospective cohort study. BMC Cardiovasc. Disord..

[5] Bian X., He J., Zhang R., Yuan S., Dou K. (2023). The Combined Effect of Systemic Immune-Inflammation Index and Type 2 Diabetes Mellitus on the Prognosis of Patients Undergoing Percutaneous Coronary Intervention: A Large-Scale Cohort Study. J. Inflamm. Res..

[6] Caiazzo G., Oliva A., Testa L., Heang T.M., Lee C.Y., Milazzo D., Stefanini G., Pesenti N., Mangieri A., Colombo A. (2024). Sirolimus-coated balloon in all-comer population of coronary artery disease patients: The EASTBOURNE DIABETES prospective registry. Cardiovasc. Diabetol..

[7] Maurina M., Chiarito M., Leone P.P., Testa L., Montorfano M., Reimers B., Esposito G., Monti F., Ferrario M., Latib A. (2023). Randomized clinical trial of abluminus DES+ sirolimus-eluting stent versus everolimus-eluting DES for percutaneous coronary intervention in patients with diabetes mellitus: An optical coherence tomography study. Catheter. Cardiovasc. Interv..

[8] Kim H., Kang D.Y., Ahn J.M., Lee J., Choi Y., Hur S.H., Park H.J., Tresukosol D., Kang W.C., Kwon H.M. (2023). Everolimus-Eluting Stents or Bypass Surgery for Multivessel Disease in Diabetics: The BEST Extended Follow-Up Study. JACC Cardiovasc. Interv..

[9] Ning C., Ling F., Liu D., Zhi Z. (2024). Ticagrelor monotherapy after a short course of dual antiplatelet therapy with ticagrelor plus aspirin following percutaneous coronary intervention in patients with versus without diabetes mellitus: A meta-analysis of randomized trials. BMC Cardiovasc. Disord..

[10] Khan F.R., Nawaz T., Sajjad W., Hussain S., Amin M., Ali H. (2024). Evaluating the Differential Risk of Contrast-Induced Nephropathy Among Diabetic and Non-diabetic Patients Following Percutaneous Coronary Intervention. Cureus.

[11] Sun S., Wang Y., Pang S., Wu X. (2024). Combination of the glycated hemoglobin levels and prognostic nutritional index as a prognostic marker in patients with acute coronary syndrome and type 2 diabetes mellitus. Lipids Health Dis..

[12] Kultursay B., Yilmaz C., Guven B., Mutlu D., Karagoz A. (2024). Potential renoprotective effect of SGLT2 inhibitors against contrast-induced AKI in diabetic STEMI patients undergoing primary PCI. Kardiol. Pol..

[13] Gaba P., Sabik J.F., Murphy S.A., Bellavia A., O’Gara P.T., Smith P.K., Serruys P.W., Kappetein A.P., Park S.J., Park D.W. (2024). Percutaneous Coronary Intervention Versus Coronary Artery Bypass Grafting in Patients with Left Main Disease with or without Diabetes: Findings From a Pooled Analysis of 4 Randomized Clinical Trials. Circulation.

[14] Byrne R.A., Rossello X., Coughlan J.J., Barbato E., Berry C., Chieffo A., Claeys M.J., Dan G.A., Dweck M.R., Galbraith M. (2023). 2023 ESC Guidelines for the management of acute coronary syndromes. Eur. Heart J..

[15] Tyczyński M., Kern A., Buller P., Wańha W., Gil R.J., Bil J. (2023). Clinical Outcomes and Prognostic Factors in Complex, High-Risk Indicated Procedure (CHIP) and High-Bleeding-Risk (HBR) Patients Undergoing Percutaneous Coronary Intervention with Sirolimus-Eluting Stent Implantation: 4-Year Results. J. Clin. Med..

[16] Tyczynski M., Kern A., Buller P., Gil R.J., Bil J. (2023). 48-Month Clinical Outcomes and Prognostic Factors in an All-Comers Population with Acute Coronary Syndrome and Chronic Coronary Syndrome Undergoing Percutaneous Coronary Intervention with a Sirolimus-Eluting Stent. J. Pers. Med..

[17] Buszman P.P., Michalak M.J., Pruski M., Fernandez C., Jelonek M., Janas A., Savard C., Gwiazdowska-Nowotka B., Zurakowski A., Wojakowski W. (2016). Comparable vascular response of a new generation sirolimus eluting stents when compared to fluoropolymer everolimus eluting stents in the porcine coronary restenosis model. Cardiol. J..

[18] Dobrolinska M., Gasior P., Roleder T., Roleder-Dylewska M., Smolka G., Ochala A., Kedhi E., Wojakowski W. (2020). Short-term healing response after implantation of the thin-strut, fast-releasing sirolimus-eluting biodegradable polymer-coated Alex Plus stent: Optical coherence tomography study. Postepy Kardiol. Interwencyjnej.

[19] Ryan T.J., Faxon D.P., Gunnar R.M., Kennedy J.W., King S.B., Loop F.D., Peterson K.L., Reeves T.J., Williams D.O., Winters W.L. (1988). Guidelines for percutaneous transluminal coronary angioplasty. A report of the American College of Cardiology/American Heart Association Task Force on Assessment of Diagnostic and Therapeutic Cardiovascular Procedures (Subcommittee on Percutaneous Transluminal Coronary Angioplasty). Circulation.

[20] Farooq V., van Klaveren D., Steyerberg E.W., Meliga E., Vergouwe Y., Chieffo A., Kappetein A.P., Colombo A., Holmes D.R., Mack M. (2013). Anatomical and clinical characteristics to guide decision making between coronary artery bypass surgery and percutaneous coronary intervention for individual patients: Development and validation of SYNTAX score II. Lancet.

[21] Lancellotti P., Zamorano J., Habib G., Badano L. (2016). The EACVI Textbook of Echocardiography.

[22] Tyczyński M., Kern A., Buller P., Gil R., Bil J. (2023). The Impact of Complete Blood Count-Derived Indices (RDW, PDW and NLR) on 4 Years Outcomes in Patients after PCI with Sirolimus-Eluting Stent, including Complex High-Risk Index Procedure (CHIP) Patients. Med. Res. J..

[23] Costa H., Espirito-Santo M., Bispo J., Guedes J., Mimoso J., Palmeiro H., Baptista Goncalves R., Vinhas H. (2023). Clinical outcomes of percutaneous coronary intervention in chronic total occlusion in patients with type 2 diabetes mellitus. Rev. Port. Cardiol..

[24] Yang Y., Hyun J., Lee J., Kim J.H., Lee J.B., Kang D.Y., Lee P.H., Ahn J.M., Park D.W., Park S.J. (2021). Effectiveness and Safety of Contemporary Drug-Eluting Stents in Patients With Diabetes Mellitus. JACC Asia.

[25] Tarantini G., Cardaioli F., De Iaco G., Tuccillo B., De Angelis M.C., Mauro C., Boccalatte M., Trivisonno A., Ribichini F., Vadala G. (2023). A more-Comers populAtion trEated with an ultrathin struts polimer-free Sirolimus stent: An Italian post-maRketing study (the CAESAR registry). Front. Cardiovasc. Med..

[26] Rozemeijer R., Benedetto D., Kraaijeveld A.O., Voskuil M., Stein M., Timmers L., Rittersma S.Z., Agostoni P., Doevendans P.A., Stella P.R. (2018). Clinical outcomes of complex real-world diabetic patients treated with amphilimus sirolimus-eluting stents or zotarolimus-eluting stents: A single-center registry. Cardiovasc. Revasc Med..

[27] Krackhardt F., Waliszewski M., Rischner J., Piot C., Pansieri M., Ruiz-Poveda F.L., Boxberger M., Noutsias M., Rios X.F., Kherad B. (2019). Nine-month clinical outcomes in patients with diabetes treated with polymer-free sirolimus-eluting stents and 6-month vs. 12-month dual-antiplatelet therapy (DAPT). Herz.

[28] Hansen K.N., Maeng M., Raungaard B., Engstrom T., Veien K.T., Kristensen S.D., Ellert-Gregersen J., Jensen S.E., Junker A., Kahlert J. (2022). Impact of diabetes on 1-year clinical outcome in patients undergoing revascularization with the BioFreedom stents or the Orsiro stents from the SORT OUT IX trial. Catheter. Cardiovasc. Interv..

[29] Babu Pothineni R., Ajmera P., Chawla K.K., Mantravadi S.S., Pathak A., Inamdar M.K., Jariwala P.V., Vijan V., Vijan V., Potdar A. (2023). Ultrathin Strut Biodegradable Polymer-Coated Sirolimus-Eluting Coronary Stents: Patient-Level Pooled Analysis From Two Indian Registries. Cureus.

[30] Su H., Cao Y., Chen Q., Ye T., Cui C., Chen X., Yang S., Qi L., Long Y., Xiong S. (2023). The association between fibrinogen levels and severity of coronary artery disease and long-term prognosis following percutaneous coronary intervention in patients with type 2 diabetes mellitus. Front. Endocrinol..

[31] Zhao C., Liu T., Wei H., Li J. (2023). Serum oxidative stress factors predict myocardial ischemia reperfusion injury after percutaneous coronary intervention in patients with acute myocardial infarction and type 2 diabetes mellitus. Postepy Kardiol. Interwencyjnej.

[32] Hemetsberger R., Mankerious N., Toelg R., Abdelghani M., Farhan S., Garcia-Garica H.M., Allali A., Windecker S., Lefevre T., Saito S. (2023). Patients with higher-atherothrombotic risk vs. lower-atherothrombotic risk undergoing coronary intervention with newer-generation drug-eluting stents: An analysis from the randomized BIOFLOW trials. Clin. Res. Cardiol..

[33] Jolly S.S., Yusuf S., Cairns J., Niemela K., Xavier D., Widimsky P., Budaj A., Niemela M., Valentin V., Lewis B.S. (2011). Radial versus femoral access for coronary angiography and intervention in patients with acute coronary syndromes (RIVAL): A randomised, parallel group, multicentre trial. Lancet.

[34] Waltenberger J., Brachmann J., van der Heyden J., Richardt G., Frobert O., Seige M., Friedrich G., Erglis A., Winkens M., Hegeler-Molkewehrum C. (2020). Five-Year Results of the Bioflow-III Registry: Real-World Experience with a Biodegradable Polymer Sirolimus-Eluting Stent. Cardiovasc. Revasc Med..

[35] Wang H., Zu Q., Tang H., Lu M., Chen R., Yang Z. (2023). Long-term cardiovascular outcomes of biodegradable polymer drug eluting stents in patients with diabetes versus non-diabetes mellitus: A meta-analysis. Cardiovasc. Diabetol..

[36] Gasior P., Gierlotka M., Szczurek-Katanski K., Osuch M., Roleder M., Hawranek M., Wojakowski W., Polonski L. (2021). Biodegradable polymer-coated thin strut sirolimus- -eluting stent versus durable polymer-coated everolimus-eluting stent in the diabetic population. Cardiol. J..

[37] Olesen K.K., Tilsted H.H., Jensen L.O., Kaltoft A., Krusell L.R., Ravkilde J., Christiansen E.H., Madsen M., Thayssen P., Sorensen H.T. (2015). Long-term outcome of sirolimus-eluting and zotarolimus-eluting coronary stent implantation in patients with and without diabetes mellitus (a Danish organization for randomized trials on clinical outcome III substudy). Am. J. Cardiol..

[38] Iglesias J.F., Heg D., Roffi M., Tuller D., Lanz J., Rigamonti F., Muller O., Moarof I., Cook S., Weilenmann D. (2019). Five-Year Outcomes in Patients With Diabetes Mellitus Treated With Biodegradable Polymer Sirolimus-Eluting Stents Versus Durable Polymer Everolimus-Eluting Stents. J. Am. Heart Assoc..

[39] Koch T., Lenz T., Joner M., Xhepa E., Koppara T., Wiebe J., Coughlan J.J., Aytekin A., Ibrahim T., Kessler T. (2021). Ten-year clinical outcomes of polymer-free versus durable polymer new-generation drug-eluting stent in patients with coronary artery disease with and without diabetes mellitus: Results of the Intracoronary Stenting and Angiographic Results: Test Efficacy of Sirolimus- and Probucol- and Zotarolimus-Eluting Stents (ISAR-TEST 5) trial. Clin. Res. Cardiol..

[40] Bruno F., Kang J., Elia E., Han J.K., De Filippo O., Yang H.M., Gallone G., Park K.W., De Luca L., Kang H.J. (2023). Impact of diabetes on long-term outcomes of bifurcation percutaneous coronary intervention. An analysis from the BIFURCAT registry. Catheter. Cardiovasc. Interv..

[41] Zibaeenezhad M.J., Sayadi M., Mohammadi S.S., Khorshidi S., Hadiyan E., Rasouli N., Karimi-Akhormeh A., Razeghian-Jahromi I. (2022). The Impact of Diabetes Mellitus on Clinical Outcomes after Percutaneous Coronary Intervention with Different Stent Sizes. J. Tehran Heart Cent..

