# Supplementary material for: Percutaneous Coronary Interventions with Sirolimus-Eluting Alex Plus Stents in Patients with or without Diabetes: 4-Year Results
Source: J Cardiovasc Dev Dis. 2024 May 22;11(6):160. doi: 10.3390/jcdd11060160 (PMC11204307; doi:10.3390/jcdd11060160)
Supplement: Supplementary file 1 [file jcdd-11-00160-s001.zip › jcdd-2960785-supplementary.pdf]

Supplementary Table S1. Univariable Cox regression for MACE

Characteristic	N	HR[1]	95% CI[1]	p-Value
Sex	97			
Male		—	—	
Female		1.01	0.41, 2.45	0.987
Age	97	1.02	0.98, 1.06	0.424
CHIP	97	2.01	0.87, 4.64	0.103
HBR	97	2.64	1.12, 6.23	0.027
ACS	97	0.71	0.31, 1.65	0.429
LM	97	3.59	0.84, 15.4	0.084
stent_length	97	1.02	0.99, 1.05	0.157
stent_diameter	97	1.17	0.53, 2.58	0.693
Lesion type	97			
A/B1		—	—	
B2/C		1.63	0.71, 3.77	0.252
Calcification	97	2.46	0.91, 6.64	0.075
second_stent	97	1.78	0.78, 4.07	0.169
MV_predilat	97	1.93	0.72, 5.19	0.194
MV_postdilat	97	2.29	1.01, 5.20	0.047
SYNTAX	84			
< 23		—	—	
23-33		2.62	0.84, 8.14	0.096
>= 33		0.00	0.00, Inf	0.998
SYNTAX_II_PCI	83			
<= 21.5		—	—	
21.5-30.6		0.51	0.10, 2.54	0.413
>= 30.6		0.63	0.17, 2.30	0.486
SYNTAX_II_CABG	75			
<= 21.5		—	—	
21.5-30.6		0.77	0.25, 2.39	0.653
>= 30.6		0.80	0.27, 2.37	0.681
Euroscore_II	97			
< 3		—	—	
3-5		3.35	1.19, 9.44	0.022
>= 5		4.74	1.82, 12.3	0.001
cardiogenic_shock	97	14.1	3.12, 63.6	<0.001
HTN	97	0.29	0.07, 1.26	0.100
dyslipidemia	97	1.18	0.35, 3.98	0.786
MI	97	2.04	0.81, 5.19	0.132
PCI	97	2.08	0.77, 5.59	0.149
CABG	97	3.24	1.33, 7.89	0.010
AO	97	2.25	0.77, 6.63	0.140
stroke	97	1.28	0.30, 5.48	0.736
smoke	97	0.90	0.40, 2.03	0.792
kidney	97	2.65	1.15, 6.14	0.022
COPD	97	2.43	0.83, 7.15	0.106
medicine_klopidogrel	97	0.37	0.13, 1.09	0.072
medicine_prasugrel	97	16.3	2.00, 133	0.009
medicine_tikagrelor	97	2.01	0.60, 6.76	0.260
medicine_ACEI	97	2.74	0.64, 11.7	0.173
medicine_ARB	97	0.20	0.03, 1.49	0.117
medicine_betabloker	97	0.01	0.00, 0.17	0.001
medicine_digoxin	97	2.43	0.33, 18.1	0.385
medicine_Ca_bloker	97	0.73	0.29, 1.85	0.503
medicine_diuretyk	97	2.63	0.90, 7.74	0.078
medicine_MRA	97	2.42	0.99, 5.90	0.052
medicine_NTG	97	0.80	0.19, 3.42	0.766
medicine_alfa_adrenolityk	97	1.62	0.48, 5.47	0.435
medicine_iwabradyna	97	4.42	0.59, 33.0	0.148

Supplementary Table S1. Univariable Cox regression for MACE

Characteristic	N	HR[1]	95% CI[1]	p-Value
medicine_acenokumarol_warafaryna	97	1.47	0.35, 6.29	0.601
medicine_rywaroksaban	97	1.77	0.41, 7.54	0.441
medicine_HDCz	97	2.17	0.51, 9.27	0.296
medicine_IPP	97	1.19	0.40, 3.48	0.758
medicine_hypoglycaemic	97	1.37	0.56, 3.33	0.488
medicine_insulin	97	0.77	0.30, 1.95	0.578
echo_EF	80	0.97	0.93, 1.00	0.077

[1]HR = Hazard Ratio, CI = Confidence Interval

Supplementary Table S2. Univariable Cox regression for TLR

Characteristic	N	HR[1]	95% CI[1]	p-Value
Sex	97			
Male		—	—	
Female		1.85	0.57, 6.07	0.308
Age	97	0.98	0.93, 1.04	0.609
CHIP	97	3.38	0.90, 12.7	0.072
HBR	97	1.17	0.36, 3.83	0.797
ACS	97	1.20	0.32, 4.51	0.791
LM	97	3.82	0.49, 29.9	0.202
stent_length	97	1.05	1.02, 1.09	0.004
stent_diameter	97	2.00	0.67, 5.96	0.214
Lesion type	97			
A/B1		—	—	
B2/C		10.3	1.32, 80.5	0.026
Calcification	97	3.16	0.83, 11.9	0.090
second_stent	97	2.36	0.69, 8.08	0.170
MV_predilat	97	5.33	0.68, 41.6	0.111
MV_postdilat	97	9.09	1.96, 42.1	0.005
SYNTAX	84			
< 23		—	—	
23-33		2.57	0.52, 12.7	0.249
>= 33		0.00	0.00, Inf	0.998
SYNTAX_II_PCI	83			
<= 21.5		—	—	
21.5-30.6		0.52	0.07, 3.66	0.507
>= 30.6		0.38	0.07, 2.07	0.262
SYNTAX_II_CABG	75			
<= 21.5		—	—	
21.5-30.6		0.77	0.16, 3.81	0.747
>= 30.6		0.92	0.20, 4.09	0.908
Euroscore_II	97			
< 3		—	—	
3-5		2.92	0.70, 12.3	0.143
>= 5		3.35	0.80, 14.1	0.098
cardiogenic_shock	97	13.1	1.56, 110	0.018
HTN	97	0.30	0.04, 2.38	0.256
Dyslipidemia	97	1.80	0.23, 14.1	0.576
MI	97	3.09	0.67, 14.3	0.149
PCI	97	1.46	0.39, 5.49	0.579
CABG	97	2.90	0.77, 10.9	0.116
AO	97	2.27	0.49, 10.5	0.294
stroke	97	1.58	0.20, 12.3	0.664
smoke	97	0.70	0.21, 2.30	0.559
kidney	97	1.63	0.43, 6.15	0.471
COPD	97	1.06	0.14, 8.25	0.959
medicine_klopidogrel	97	0.22	0.06, 0.83	0.025
medicine_prasugrel	97	29.3	3.21, 268	0.003
medicine_tikagrelor	97	2.86	0.62, 13.3	0.179
medicine_Ca_bloker	97	1.69	0.52, 5.55	0.385
medicine_diuretyk	97	1.47	0.39, 5.53	0.572
medicine_MRA	97	1.15	0.25, 5.33	0.858
medicine_NTG	97	0.83	0.11, 6.51	0.862
medicine_alfa_adrenolityk	97	2.32	0.50, 10.8	0.282
medicine_IPP	97	1.16	0.25, 5.38	0.847
medicine_hypoglycaemic	97	0.99	0.29, 3.38	0.986
medicine_insulina	97	0.49	0.11, 2.26	0.358
echo_EF	80	1.06	0.96, 1.17	0.278

[1]HR = Hazard Ratio, CI = Confidence Interval

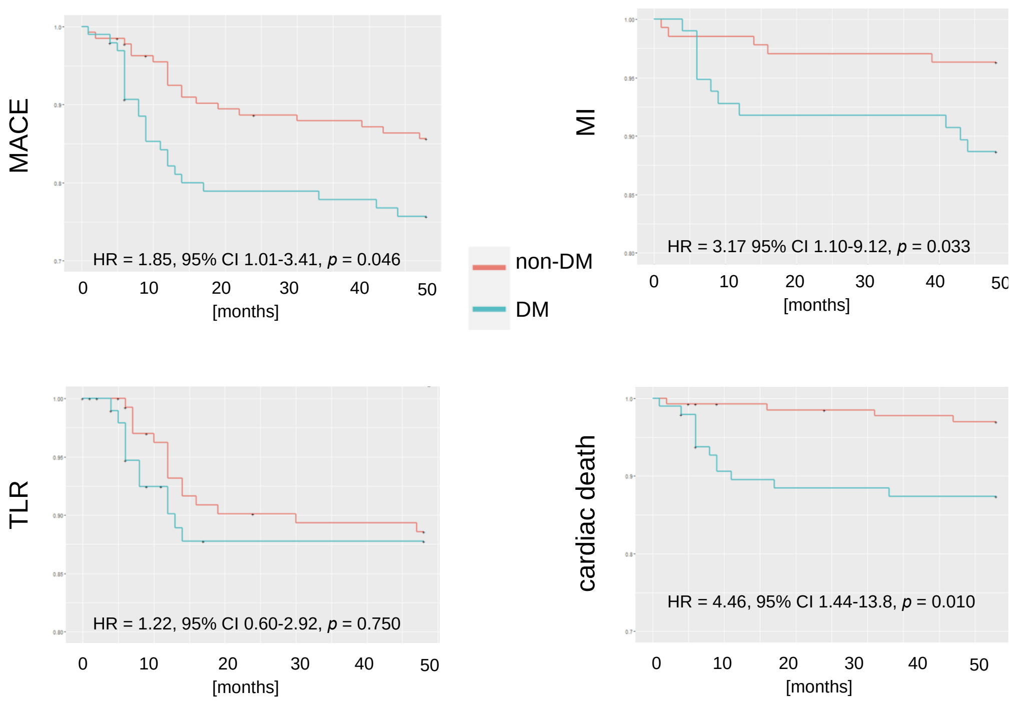

Supplementary Figure S1. Kaplan-Meier curves disclosing event-free survival in DM and non-DM subgroups. DM - diabetes; MACE – major adverse cardiovascular events; MI – myocardial infarction; TLR – target lesion revascularization

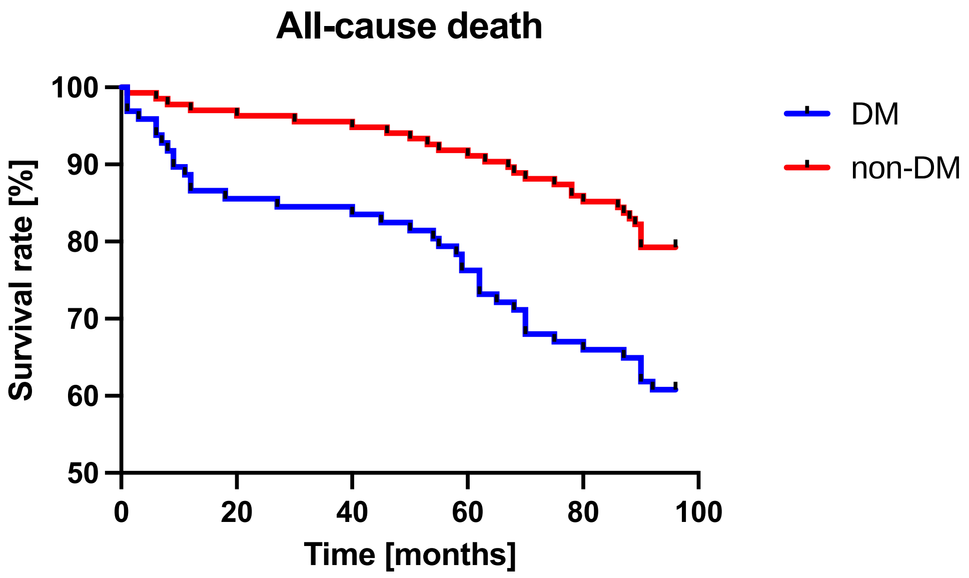

Supplementary Figure S2. Kaplan-Meier curves disclosing event-free survival in DM and non-DM subgroups in the whole population at 8 years. DM - diabetes; MACE – major adverse cardiovascular events; MI – myocardial infarction; TLR – target lesion revascularization
